# Use of a design challenge to develop postural support devices for intermediate wheelchair users

**DOI:** 10.4102/ajod.v6i0.346

**Published:** 2017-09-08

**Authors:** Brenda N. Onguti, Deepti Tanuku, Elizabeth J. Himelfarb Hurwitz, Nathaniel C. Moller, Youseph Yazdi, Shannon Egan, Eva S. Bazant, Anthony Gichangi

**Affiliations:** 1 Innovations Unit, Jhpiego, Johns Hopkins University, United States; 2 Johns Hopkins Center for Bioengineering Innovation & Design (CBID), Johns Hopkins University, United States; 3 Monitoring Evaluation Research Unit, Jhpiego, Johns Hopkins University, United States; 4 Monitoring Evaluation Research Unit, Jhpiego, Johns Hopkins University, Kenya

## Abstract

The provision of an appropriate wheelchair, one that provides proper fit and postural support, promotes wheelchair users’ physical health and quality of life. Many wheelchair users have postural difficulties, requiring supplemental postural support devices for added trunk support. However, in many low- and middle-income settings, postural support devices are inaccessible, inappropriate or unaffordable. This article describes the use of the design challenge model, informed by a design thinking approach, to catalyse the development of an affordable, simple and robust postural support device for low- and middle-income countries. The article also illustrates how not-for-profit organisations can utilise design thinking and, in particular, the design challenge model to successfully support the development of innovative solutions to product or process challenges.

## Introduction

Globally, an estimated 70 million people require wheelchairs (World Health Organization [WHO] n.d.). When a wheelchair user is equipped with an appropriately fitting wheelchair that provides postural support, it promotes physical well-being and improves quality of life. For wheelchair users, better posture means greater comfort, enhanced safety, improved breathing and digestion, and greater mobility (WHO & USAID 2013). Wheelchair users who have good trunk strength and stability can independently sit upright when provided with a basic postural support system which includes the backrest, cushion, footrests and armrests of a wheelchair (WHO, ISPO & USAID 2008). However, many wheelchair users have postural difficulties and require supplemental postural support devices for upright seating. Postural support devices brace the wheelchair user’s body in an upright position when they are unable to do so independently. The design of postural support devices varies depending on the support they are intended to provide; different devices are used to provide stability to the pelvis, hips, trunk, head, thighs or lower legs (WHO & USAID 2013).

As few as 5% of persons in need of properly fitted wheelchairs have access to one (WHO & USAID 2012). Globally, the lack of access and availability disproportionately affects wheelchair users in low- and middle-income countries. A number of reasons contribute to this: wheelchairs that have integrated postural support devices are more expensive and are difficult to obtain. Additive postural support devices are not standardised across manufacturers or wheelchair style and cannot be effectively interchanged between wheelchairs – for example, when a user acquires a new wheelchair. Poor wheelchair fit is one of the contributors to wheelchair abandonment and underutilisation in less-resourced settings (Mukherjee & Samanta 2005). Postural support devices therefore need to be redesigned for use in low- and middle-income countries to ensure availability, accessibility and suitability for individuals living with mobility impairments.

## What is a design challenge?

A design challenges is an innovation competition or collaboration that focuses on quickly generating product or process designs to meet the specific needs of particular end users (Design Council n.d.). A design challenge draws on design thinking, a problem solving methodology that encourages rapid prototyping, iteration, and learning, to help propel innovators past common design roadblocks and prioritises product suitability and usability by providing structured opportunities for stakeholder feedback. When properly implemented, design thinking disrupts thinking based on conventional biases, like an inclination to one’s own view of a problem and its solution or an end users’ inability to describe their need (Jeanne 2015). Therefore, value is placed on developing a comprehensive understanding of the needs of stakeholders thus reframing design obstacles to yield solutions with lasting impact (IDEO.org 2015).

### A design challenge for wheelchair postural support devices

Accelovate, a United States Agency for International Development-funded programme led by Jhpiego in Baltimore, MD, United States, hosted a design challenge to catalyse the design of postural support devices suitable for and desirable to end users in low- and middle-income countries.

Innovators from around the world were provided with seed funding, technical assistance and peer review to guide, support and accelerate the design and early-stage commercialisation of high-quality postural support devices for less-resourced settings. Accelovate’s design challenge was conducted in three phases: (1) identification of need, (2) concept development and iteration and (3) transition to commercialisation.

#### Phase I: Identification of need

The Accelovate team consulted with wheelchair and disability experts to better understand the current challenges faced by those living with mobility impairments in less-resourced settings. The experts confirmed that a more appropriately designed postural support device – one that is sturdy, affordable, locally repairable and useable across a wide variety of wheelchairs – would make a tremendous improvement in wheelchair users’ lives in low- and middle-income countries.

Incorporating the feedback from experts, the design challenge focused on functional, market-ready postural support device prototypes. By defining the challenge and sharing findings among potential innovators, the Accelovate programme reduced information barriers to effective problem solving and created an environment for the efficient use of resources. Armed with high-quality information from disability experts, innovators could move directly into the concept generation and development stage without spending resources on redundant user and market research.

#### Phase II: Concept development and iteration

The design challenge convening organisation is not typically the key innovator; instead, the convening organisation facilitates competition, collaboration and cross-pollination of ideas and insights across teams of innovators.

To ensure the process generated contextually appropriate designs, Accelovate encouraged and prioritised designers and innovators in less-resourced settings. In an effort to diversify perspectives, Accelovate also targeted innovators outside the mobility sector, including universities, non-governmental organisations, faith-based organisations and for-profit partners. Through email blasts, social media and conference presentations, the Accelovate design challenge reached more than 100 000 people from 32 countries.

The design challenge technical review committee, with a broad range of expertise, evaluated the preliminary proposals and prototype submissions, facilitated the development of the design challenge process, developed selection criteria and mentored innovation teams. Furthermore, the technical review committee provided support in areas where the innovators lacked expertise. The multidisciplinary team included experts in business, engineering, clinical practice and public health; their varied perspectives prompted innovators to consider diverse product development and market introduction factors.

In total, 12 concept notes were submitted for consideration. The technical review committee winnowed submissions and the six that were determined to have the highest potential marketability, sustainability, and usability were selected. These six teams were awarded small seed grants to support further development of their designs and to build initial product prototypes.

Design challenges encourage collaboration through competition. Rival innovators compete to develop the best solution to a problem. Once the initial prototypes were complete, the teams were invited to Washington, DC, to present to the review committee and the other innovators. After evaluation by the review committee, the teams also participated in a peer review process. The three prototypes considered most desirable, technologically feasible and commercially viable were selected to receive additional funding and technical support to develop and test final prototypes.

Opportunities for collaboration and co-creation were critical for the development of the final postural support device prototypes. These opportunities encouraged competitors to support each other and share information and ideas; in this way, all participants, even those that were not ultimately designated as the top teams, benefited from participation.

#### Phase III: Transition to commercialisation

Accelovate facilitated connections between the innovators and important potential donors, distributors and purchasers within the disability and mobility sector. Throughout the Accelovate design challenge, innovators were encouraged to actively seek feedback from stakeholders and end users with specialised expertise in low- and middle-income health systems.

During the last phase of the design challenge, innovation teams were required to develop commercialisation and implementation strategies. Each team was prompted to evaluate their target markets – focusing on stakeholder dynamics, barriers to entry and risk mitigation factors – to facilitate the development of robust business plans for introduction and product adoption.

Two postural support device prototypes that were developed during the Accelovate design challenge have since moved to commercial production and are being implemented and used in east Africa and India. Following the design challenge, one of the finalist innovation teams elected to purchase a unique component from a rival participant to further enhance their product. This collaborative relationship between rival teams, resulting in the production of a potentially superior end product, is a major benefit of the design challenge.

## Discussion

The objective of this article was to highlight how design challenge, a tool in the design thinking toolbox, was utilised to catalyse the design of affordable, simple and robust postural support devices for the low-resource settings.

### Limitations of the design challenge

A limitation of the design challenge is that if awareness of the design challenge process fails to reach the right organisations, the best organisations may not apply. To mitigate this, Accelovate made significant efforts to disseminate the request for applications to ensure that innovators from around the world had the opportunity to participate.

Another potential limitation is that the quality of the marketable product is dependent on the innovative team. Without specific standards and ways of measurement, a design challenge may not yield the intended outcome. Accelovate countered this through having a rigorous selection criterion on what concept and prototypes got funded. Accelovate provided constant support to the teams awarded a sub-grant via relevant theme focused webinars, mentorship from technical review committee members and virtual collaboration sessions with other teams.

Funders of an organisation holding the design challenge may have certain restrictions on who can apply to participate. These restrictions decrease the pool of applicants and potentially lock out some competitive innovative teams.

Lastly, though it was hoped that innovative teams from diverse social and commercial sectors would apply, only those in the wheelchair sector actually did. This may have resulted from the problem statement not appealing to those outside the sector. The contribution of outside organisations was unknown in this case.

## Conclusion

Too often, innovations flounder during market introduction because designers and developers fail to consider the context in which their products will function and the full range of stakeholder and user perspectives.

Design thinking prompts innovators to challenge their assumptions early and often by continuously seeking feedback from key stakeholders, including end users, service providers, distributors or manufacturers. This helps innovators to share new perspectives and ideas. This new perspective reframes product and process development, emphasising the core needs of end users, market dynamics and the product environment as fundamental considerations of early-stage product design.

The design challenge offers an ideal product development framework that can be utilised in low- and middle-income settings. It is a model for lean innovation; it encourages the efficient use of resources and prioritises product suitability and sustainability. The Accelovate design challenge illustrates how not-for-profit organisations can successfully support the development of innovative solutions to product or process challenges by focusing on the user and other stakeholders as a means to efficiently develop viable innovations. The design challenge – and design thinking more generally – provides a robust product and process design platform for donors, governments and implementing organisations and should be considered in other sectors focused on low- and middle-income settings.
